# A Retrospective Analysis of 10-Year Data Assessed the Diagnostic Accuracy and Efficacy of Cytogenomic Abnormalities in Current Prenatal and Pediatric Settings

**DOI:** 10.3389/fgene.2019.01162

**Published:** 2019-11-20

**Authors:** Hongyan Chai, Autumn DiAdamo, Brittany Grommisch, Fang Xu, Qinghua Zhou, Jiadi Wen, Maurice Mahoney, Allen Bale, James McGrath, Michele Spencer-Manzon, Peining Li, Hui Zhang

**Affiliations:** ^1^Department of Genetics, Yale University School of Medicine, New Haven, CT, United States; ^2^Prevention Genetics, Marshfield, WI, United States; ^3^The First Affiliated Hospital, Biomedical Translational Research Institute, Jinan University, Guangzhou, China

**Keywords:** prenatal and pediatric diagnosis, chromosomal abnormalities, recurrent genomic disorders, microdeletions and microduplications, pathogenic copy number variants, abnormality detection rate, relative frequency, diagnostic accuracy and efficacy

## Abstract

**Background:** Array comparative genomic hybridization (aCGH), karyotyping and fluorescence *in situ* hybridization (FISH) analyses have been used in a clinical cytogenetic laboratory. A systematic analysis on diagnostic findings of cytogenomic abnormalities in current prenatal and pediatric settings provides approaches for future improvement.

**Methods:** A retrospective analysis was performed on abnormal findings by aCGH, karyotyping, and FISH from 3,608 prenatal cases and 4,509 pediatric cases during 2008–2017. The diagnostic accuracy was evaluated by comparing the abnormality detection rate (ADR) and the relative frequency (RF) of different types of cytogenomic abnormalities between prenatal and pediatric cases. A linear regression correlation between known prevalence and ADR of genomic disorders was used to extrapolate the prevalence of other genomic disorders. The diagnostic efficacy was estimated as percentage of detected abnormal cases by expected abnormal cases from served population.

**Results:** The composite ADR for numerical chromosome abnormalities, structural chromosome abnormalities, recurrent genomic disorders, and sporadic pathogenic copy number variants (pCNVs) in prenatal cases were 13.03%, 1.77%, 1.69%, and 0.9%, respectively, and were 5.13%, 2.84%, 7.08%, and 2.69% in pediatric cases, respectively. The chromosomal abnormalities detected in prenatal cases (14.80%) were significantly higher than that of pediatric cases (7.97%) (p < 0.05), while the pCNVs detected in prenatal cases (2.59%) were significantly lower than that of pediatric cases (9.77%) (p < 0.05). The prevalence of recurrent genomic disorders and total pCNVs was estimated to be 1/396 and 1/291, respectively. Approximately, 29% and 35% of cytogenomic abnormalities expected from the population served were detected in current prenatal and pediatric diagnostic practice, respectively.

**Conclusion:** For chromosomal abnormalities, effective detection of Down syndrome (DS) and Turner syndrome (TS) and under detection of sex chromosome numerical abnormalities in both prenatal and pediatric cases were noted. For pCNVs, under detection of pCNVs in prenatal cases and effective detection of DiGeorge syndrome (DGS) and variable efficacy in detecting other pCNVs in pediatric cases were noted. Extend aCGH analysis to more prenatal cases with fetal ultrasonographic anomalies, enhanced non-invasive prenatal (NIPT) testing screening for syndromic genomic disorders, and better clinical indications for pCNVs are approaches that could improve diagnostic yield of cytogenomic abnormalities.

## Introduction

Cytogenetic analysis has been used in the etiological diagnosis for pediatric patients with developmental delay (DD), intellectual disability (ID), multiple congenital anomalies (MCA), and autistic spectrum disorders (ASD) and for pregnancies in risk of chromosomal abnormalities or with fetal defects (Smeets, 2004; Veltman, 2006). Chromosomal analysis has been effective in detecting numerical and structural chromosomal abnormalities on cultured cells from specimens obtained from peripheral blood (PB), amniocentesis (AC), and chorionic villus sampling (CVS) despite its analytical resolution limited by the average G-band size of 5–10 megabase (Mb). Fluorescence *in situ* hybridization (FISH) in metaphase or interphase cells using labeled DNA probes of 100–800 kilobase (Kb) has improved the analytical resolution allowing detection of the targeted microdeletions, microduplications, and rearrangements. Multiple FISH panels with targeted probes have been used to detect common aneuploidies and cryptic subtelomeric rearrangements (Ried et al., 1992; Ning et al., 1996). Conventional cytogenetic evaluation of ID/DD/MCA showed an abnormality detection rate (ADR) of 3.7% for numerical and large structural chromosomal abnormalities and up to 6.8% when combined with FISH analysis for targeted cryptic and subtelomeric rearrangements (Shaffer, 2005).

In the past decade, genome-wide microarray analysis using array comparative genomic hybridization (aCGH) or single nucleotide polymorphism (SNP) chip has been validated and applied as the first-tier genetic testing for pediatric patients with ID/DD/MCA (Xiang et al., 2008; Miller et al., 2010). The application of aCGH for large case series of newborns and pediatric patients has been reported. The ADR of cytogenomic abnormalities from pediatric patients with ID/DD/MCA and autism is in the range of 12%–20% (Xiang et al., 2010; Ahn et al., 2013; Park et al., 2013). Significantly improved diagnostic yield of pathogenic copy number variants (pCNVs) from pediatric patients has led to a rapid application of this genomic analysis for prenatal diagnosis (Wei et al., 2013; Zhou et al., 2016). Opinion and guidelines for prenatal diagnosis using microarray analysis have been introduced by the American College of Obstetricians and Gynecologists and the American College of Medical Genetics and Genomics (ACOG Committee Opinion No. 446, 2009; South et al., 2013). Integration of microarray analysis into conventional cytogenetic analysis has been effective in defining the gene contents and genomic coordinates for recurrent genomic disorders and chromosomal structural imbalances.

This report, a retrospective analysis of prenatal and pediatric cases over a 10-year interval in the Yale Clinical Cytogenetics Laboratory, has revealed the difference in diagnostic yield on the spectrum of cytogenomic abnormalities. The findings from this study provide guidance for future improvement of prenatal and pediatric diagnosis of cytogenomic abnormalities.

## Materials and Methods

### Subjects

The Yale Laboratory of Clinical Cytogenetics is CLIA-certified and provides diagnostic services for prenatal and pediatric patients. Clinical cases referred for cytogenetic analysis and test results during the interval of 2008–2017 were retrieved from the laboratory’s CytoAccess database (Xiang et al., 2006), which showed 3,608 consecutive prenatal cases and 4,509 consecutive pediatric cases (age <18 years old) for a total of 8,117 cases. Standardized karyotyping, FISH, and aCGH tests were performed for the prenatal and pediatric patients following requisitions of referring physicians. Of the 3,608 prenatal cases, 2,269 cases were analyzed by karyotyping only, 781 cases were by karyotyping and aCGH, 331 cases were by karyotyping and FISH, 185 cases were by karyotyping, aCGH, and FISH, 31 cases were by aCGH only, seven cases by aCGH and FISH, and four cases were by FISH only. Of the 4,509 pediatric cases, 1,526 cases were analyzed by aCGH only, 1,401 cases by karyotyping and aCGH, 1,091 by karyotyping only, 194 cases by karyotyping, FISH, and aCGH, 133 cases by karyotyping and FISH, 89 cases by aCGH and FISH, and 75 cases by FISH only. For this retrospective study, there were no pre-study requirements on the patient’s clinical indications and specimens and there was no post-study interaction with the patients and their families. This project was determined as a chart review retrospective study and deemed exempted from Institutional Review Board (IRB) approval and granted waiver of consent based on the policy of the Yale University IRB.

### Karyotyping, FISH, and aCGH

Karyotyping was performed on G-band metaphases prepared from cultured cells of PB, AC, and CVS following the laboratory’s standardized procedures. FISH tests using the AneuVysion probes (Abbott Inc. Des Plaines, IL) were performed for rapid detection of common aneuploidies involving chromosomes X, Y, 13, 18, and 21. FISH tests using probes for known microdeletion and microduplication loci (Cytocell Inc. Cambridge, UK) were performed for a rapid screening or confirmation.

Oligonucleotide aCGH analysis was performed on genomic DNA extracted from peripheral blood lymphocytes, directly prepared villi cells or cultured amniocytes using the Gentra Puregene Kit (Qiagen, Valencia, CA). The DNA concentration was measured using a Nano-Drop spectrophotometer (Thermo Fisher Scientific, Inc. Waltham, MA). High molecular weight DNA was verified by agarose gel electrophoresis. For each sample, 2 ug of genomic DNA was used following the manufacturer’s protocol from the Agilent Human Genome CGH microarray 180K kit (110,712 CGH + 59,647 SNP 60-mer oligonucleotides) and 400K kit (292,097 CGH + 119,091 SNP 60-mer oligonucleotide probes) (Agilent Technologies, Inc., Santa Clara, CA). This aCGH procedure can achieve 99% sensitivity and 99% specificity using a sliding window of five to seven contiguous oligonucleotides, indicating an analytical resolution of 100–150 Kb for the 180K kit and 40–60 Kb for the 400K kit (Xiang et al., 2008). The genomic coordinates for detected aberrations from this aCGH analysis were based on the February 2009 Assembly (GRCh37/hg19) of the UCSC Human Genome browser (http://genome.ucsc.edu/).

### Categorization of the Cytogenomic Abnormalities

**Figure 1:**
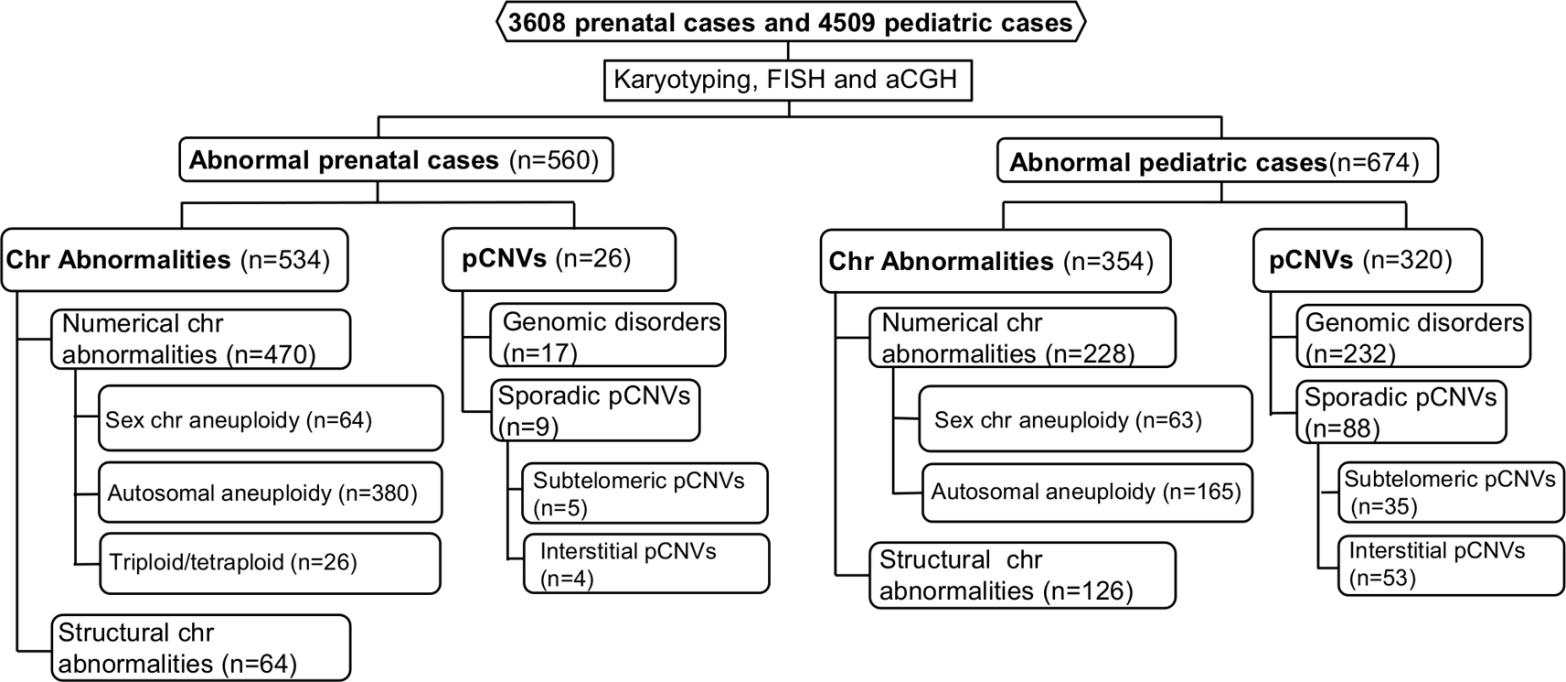
Flow-diagram for detection and categorization of cytogenomic abnormalities in prenatal and pediatric cases. aCGH, array comparative genomic hybridization; Chr, chromosome; FISH, fluorescence *in situ* hybridization; pCNVs, pathogenic copy number variants.

The spectrum of cytogenomic abnormalities was divided into two major categories: chromosomal abnormalities and submicroscopic genomic aberrations which were pCNVs that are 5–10 Mb and less. The chromosomal abnormalities were further divided into two major types: (1) numerical abnormalities including sex chromosome aneuploidies (47,XXY, 47,XYY, other X or Y aneuploidies for males and 45,X, 47,XXX, other X aneuploidies for females), autosomal aneuploidies (trisomy 21, trisomy 18, trisomy 13, and other autosomal aneuploidies), and polyploidies (triploidy and tetraploidy); and (2) structural abnormalities including balanced rearrangements (reciprocal translocations, Robertsonian translocations, inversions, etc.) and unbalanced structural rearrangements (deletions, duplications, marker chromosomes, etc.) (Nussbaum et al., 2007). Based on the American College of Medical Genetics and Genomics (ACMG) standards and guidelines, only pCNVs were included in this study (Kearney et al., 2011). The detected pCNVs were further divided into two major types: (1) recurrent genomic disorders (also termed as microdeletion and microduplication syndromes) induced by low copy repeats and segmental duplications, and (2) sporadic pCNVs detected in the subtelomeric and interstitial regions. Excluding the recurrent genomic disorders, pCNVs detected at the terminal 10–15 Mb in large autosomes numbered 1 to 12 and terminal 5–10 Mb in small autosomes numbered 13 to 22, were usually considered to be subtelomeric pCNVs; pCNVs found between centromeric and subtelomeric regions were interstitial pCNVs (Xu et al., 2014a). The workflow of prenatal and pediatric case processing with detected cytogenomic abnormalities and their categories is depicted in **Figure 1**.

### Comparison of ADR and RF From Prenatal and Pediatric Cases

To evaluate the diagnostic accuracy for various types of abnormalities in prenatal or pediatric settings, ADR in prenatal and pediatric patients was calculated by the number of abnormal cases divided by the total number of patients. Among the prenatal cases, the ADR of chromosomal abnormalities was calculated by the number of abnormal chromosomal cases divided by the total number of 3,608 prenatal cases; the ADR of pCNVs was calculated by the number of pCNVs cases divided by the number of 1,004 prenatal cases analyzed by aCGH. Among pediatric patients, the ADR of chromosomal abnormalities was calculated by the number of abnormal chromosomal cases divided by the total number of 4,443 cases, which included 4,434 cases analyzed by karyotyping or aCGH and nine FISH only cases for common aneuploidies; the ADR of pCNVs was calculated by the number of pCNVs cases divided by the total number of 3,276 cases, which included 3,210 cases analyzed by aCGH and 66 FISH only cases for specific microdeletions or microduplications. The ADR for chromosomal abnormalities and pCNVs was based on the different number of cases performed and was considered as a composite ADR.

To evaluate the spectrum of chromosomal abnormalities and pCNVs in prenatal or pediatric settings, relative frequency (RF) was determined by the number of abnormal prenatal or pediatric cases in each major type divided by the total number of chromosomal abnormalities or pCNVs in prenatal or pediatric cases, respectively. Chi-square tests were applied to compare ADR of chromosomal abnormalities and pCNVs between prenatal and pediatric cases and a P value of less than 0.05 was considered statistically significant.

### Estimating Prevalence and Diagnostic Efficacy of Cytogenomic Abnormalities

Constitutional chromosomal aneuploidies are the result of nondisjunction in meiosis in the great majority. Recurrent genomic disorders are caused by nonallelic homologous recombination (NAHR) mediated by low copy repeats or segmental duplications. Even though genomic disorders are caused by the same mechanism, prevalence of specific disorders varied from relatively common to extremely rare due to the genomic structure of low copy repeats or segmental duplications (Gillentine et al., 2018). A linear regression analysis was performed to correlate the ADR in the pediatric setting with epidemiological data for DiGeorge/velocardiofacial syndrome (DGS/VCFS, 22q11.21 deletion), Williams-Beuren syndrome (WBS, 7q11.23 deletion), Prader-Willi/Angelman syndromes (PWS/AS, 15q11-q13 deletion), Smith-Magenis syndrome (SMS, 17p11.2 deletion), and hereditary neuropathy with liability to pressure palsies (HNPP, 17p12 deletion) (Greenberg et al., 1991; Kyllerman, 1995; Meretoja et al., 1997; Strømme et al., 2002; Oiglane-Shlik et al., 2006; Shprintzen, 2008; Gillentine et al., 2018). Correlation with a P value less than 0.05 was considered statistically significant and was further used to extrapolate the prevalence of other rare genomic disorders, including recurrent genomic disorders and pCNVs. By a similar procedure, a linear correlation between the ADR of 45,X (Turner syndrome, TS), trisomy 21 (Down syndrome, DS), trisomy 18, and trisomy 13 in the pediatric setting and their incidences (Nussbaum et al., 2007) was used to estimate the diagnostic outcome of 47,XXY, 47,XYY, 47,XXX, and balanced Robertsonian translocations.

Given the known incidence of DS and DGS, the ratio of DS/DGS was used as a simple measurement of efficacy in detecting chromosome abnormalities and pCNVs. From the average annual abnormal cases of DS and DGS, the size of the population served was estimated. The expected number of abnormal cases with chromosomal abnormalities and pCNVs was calculated from known incidence or prevalence times the estimated population served. Diagnostic efficacy, defined as the likelihood of a patient with a cytogenomic abnormality being detected in current prenatal or pediatric clinical setting, was calculated by detected number of abnormal cases divided by expected number of abnormal cases.

## Results

### Prenatal and Pediatric Caseloads

**Figure 2:**
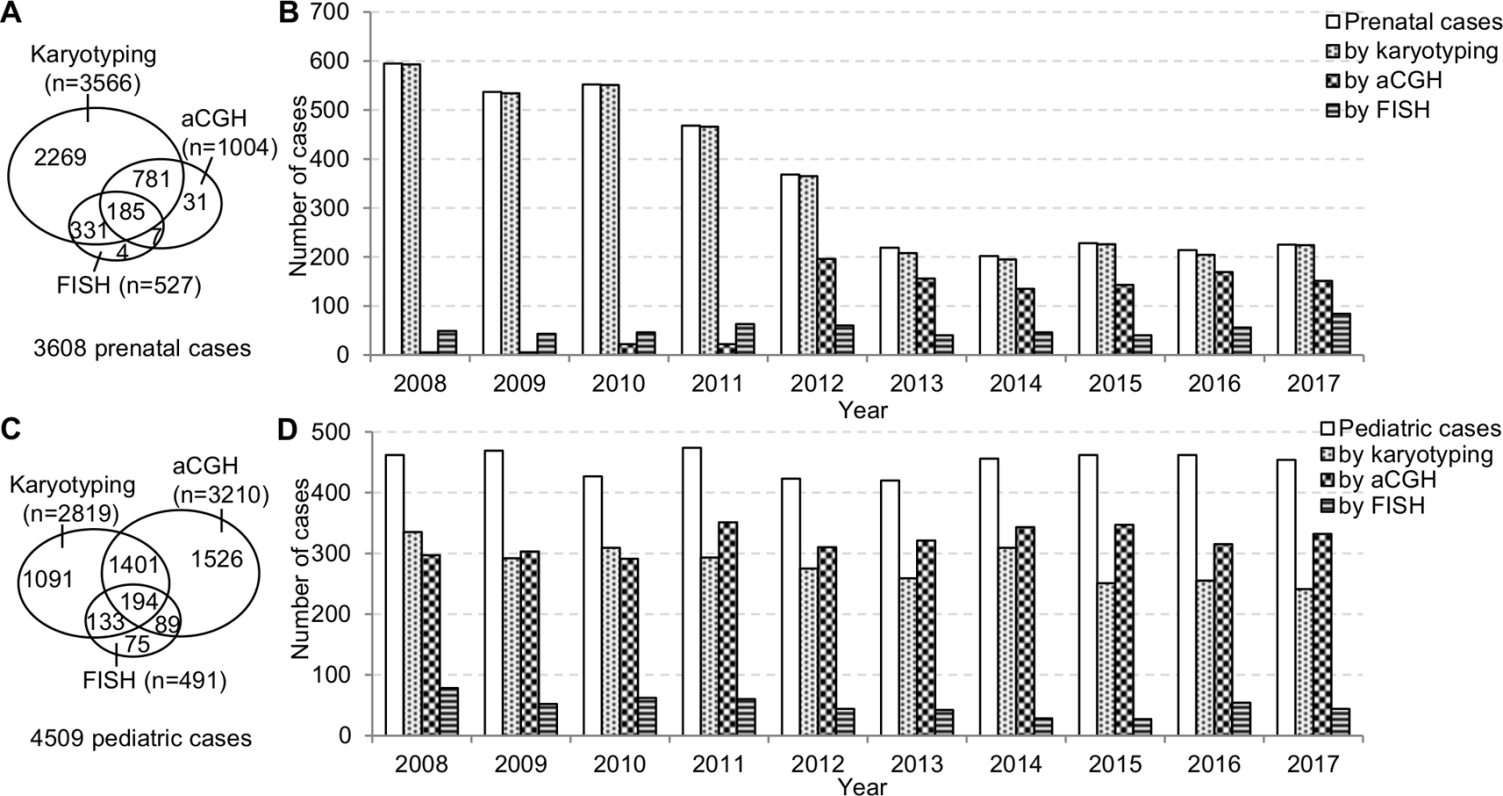
The prenatal and pediatric caseloads during 2008–2017. **(A)** The total number of cases and **(B)** the annual number of cases analyzed by karyotyping, aCGH, and FISH in the prenatal setting. **(C)** The total number of cases and **(D)** the annual number of cases analyzed by karyotyping, aCGH and FISH in the pediatric setting. aCGH, array comparative genomic hybridization; FISH, fluorescence *in situ* hybridization.

The prenatal and pediatric caseloads during 2008–2017 were shown in **Figure 2**. The annual prenatal caseload showed a decrease from 595 cases in 2008 to 219 cases in 2013, and then stabilized at around 210 cases annually from 2013 to 2017. Although aCGH had been increasingly applied to prenatal diagnosis, karyotyping was still requested in almost all prenatal cases. In the 10-year period, less than one third of total prenatal cases had aCGH. The annual pediatric caseload was relatively stable, and the most frequently applied analytical method to pediatric cases has changed from karyotyping to aCGH.

### Diagnostic Yields of Cytogenomic Abnormalities in Prenatal and Pediatric Settings

Cytogenomic abnormalities were detected in a total of 560 prenatal cases and 674 pediatric cases, and the ADR in prenatal and pediatric settings were 15.52% (560/3,608) and 14.95% (674/4,509), respectively. Details of all abnormal prenatal and pediatric cases are summarized in the supplemental files (**Tables S1**–**S4**). The ADR of chromosomal abnormalities in prenatal cases was 14.80% which was significantly higher than the ADR of 7.97% in pediatric cases (p < 0.05). The ADR of pCNVs in prenatal cases was 2.59% which was significantly lower than the ADR of 9.77% in pediatric cases (p < 0.05).

**Figure 3:**
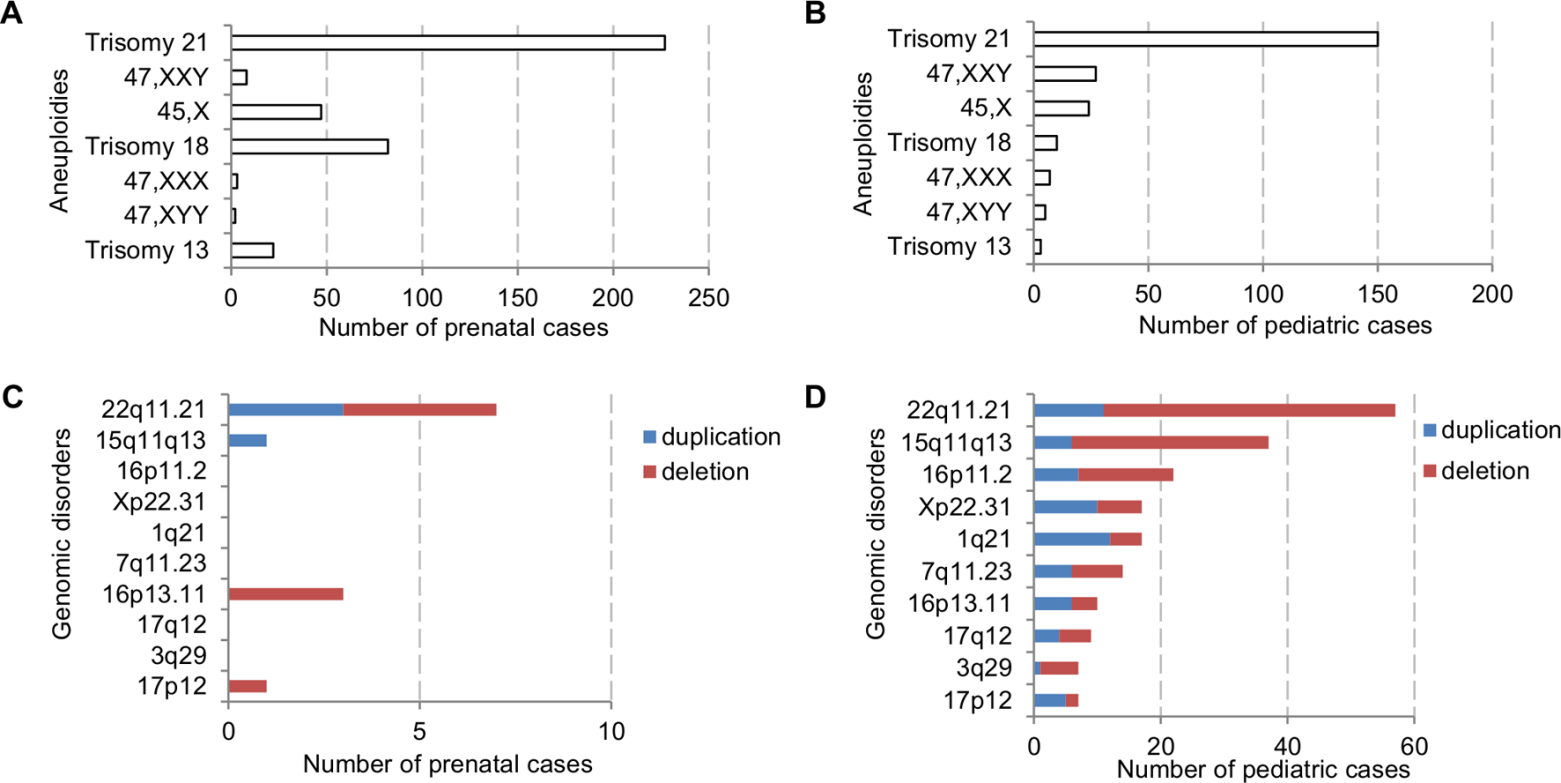
The number of cases with common chromosomal abnormalities detected in **(A)** prenatal and **(B)** pediatric settings. The number of cases with common recurrent genomic disorders detected in **(C)** prenatal and **(D)** pediatric settings.

The major types of chromosomal abnormalities in prenatal and pediatric cases are summarized in **Table 1** and **S5**. The ADR for sex chromosome aneuploidies, autosomal aneuploidies, balanced structural rearrangements, and unbalanced rearrangements were 1.77%, 10.53%, 0.91%, and 0.86% for prenatal cases, and 1.42%, 3.71%, 0.63%, and 2.21% for pediatric cases. In prenatal cases, the most frequently seen numerical chromosomal abnormalities by RF were trisomy 21, trisomy 18, 45,X, and trisomy 13 at 42.51%, 15.36%, 8.8%, and 4.12%, respectively, which accounted for 70.79% of the chromosome abnormalities. In pediatric cases, the most frequently seen numerical chromosomal abnormalities by RF were trisomy 21, 47,XXY, 45,X, and trisomy 18 at 42.37%, 7.63%, 6.78%, and 2.82%, respectively, which accounted for 59.6% of the chromosome abnormalities. **Figures 3A, B** show the number of cases of common aneuploidies detected in prenatal and pediatric cases. For structural chromosomal abnormalities, RF for balanced and unbalanced rearrangements were 6.18% and 5.81% for prenatal cases and 7.91% and 27.68% for pediatric cases, respectively.

**Table 1:** Chromosomal abnormalities detected in prenatal and pediatric settings.

Chromosomal abnormalities	Prenatal			Pediatric		
	No. Abn Cases	ADR	RF	No. Abn Cases	ADR	RF
**Numerical abnormality**						
**Sex Chromosome Aneuploidy**						
Males						
47,XXY	8	0.22	1.50	27	0.61*	7.63
47,XYY	2	0.06	0.37	5	0.11	1.41
Females						
45,X	47	1.30	8.80	24	0.54*	6.78
47,XXX	7	0.19	1.31	7	0.16	1.98
**Subtotal**	**64**	**1.77**	**11.99**	**63**	**1.42**	**17.80**
**Autosomal Aneuploidy**						
Trisomy 21	227	6.29	42.51	150	3.38*	42.37
Trisomy 18	82	2.27	15.36	10	0.23*	2.82
Trisomy 13	22	0.61	4.12	3	0.07*	0.85
Other aneuploidy	49	1.36	9.18	2	0.05*	0.56
**Subtotal**	**380**	**10.53**	**71.16**	**165**	3.71*	**46.61**
**Triploid &tetraploid**	**26**	**0.72**	**4.87**	**0**		
**Total**	**470**	**13.03**	**88.01**	**228**	5.13*	**64.41**
**Structural abnormality**						
**Balanced rearrangements**						
Robertsonian translocations	6	0.17	1.12	4	0.09	1.13
Other	27	0.75	5.06	24	0.54	6.78
**Subtotal**	**33**	**0.91**	**6.18**	**28**	**0.63**	**7.91**
**Unbalanced rearrangements**	**31**	**0.86**	**5.81**	**98**	2.21*	**27.68**
**Total**	**64**	**1.77**	**11.99**	**126**	2.84*	**35.59**
**All chromosomal abnormalities**	**534**	**14.80**	**100.00**	**354**	7.97*	**100.00**

ADR (abnormality detection rate by %): no. abn cases/3608 prenatal cases; no. abn cases/4443 pediatric cases. (4443 pediatric cases including 4434 cases analyzed by karyotyping or aCGH and 9 cases by FISH only for common aneuploidies). RF (relative frequency by %): no. abn cases/534 total prenatal chr abn; no. abn cases/354 total pediatric chr abn. *Statistically different from ADR in prenatal setting (p < 0.05).

The types of pCNVs detected in prenatal and pediatric cases are summarized in **Table 2**. The ADR for recurrent genomic disorders and sporadic pCNVs were 1.69% and 0.90% for prenatal cases, and 7.08% and 2.69% for pediatric cases, respectively. These results showed significant under detection of pCNVs in prenatal cases (P <0.05). **Figures 3C, D** show the number of cases of 10 recurrent genomic disorders most commonly detected in prenatal and pediatric cases. Only a few genomic disorders at 22q11.21, 16p13.11, 17p13.3, and 15q13 loci were detected prenatally. The most frequently seen recurrent genomic disorders by RF were microdeletions and duplications at 22q11.21, 15q11-q13, 16p11.2, Xp22.31, 1q21, 7q11.23, 16p13.11, 17q12, 3q29, and 17p12 at 17.81%, 11.56%, 6.88%, 5.31%, 4.69%, 4.38%, 3.13%, 2.81%, 2.19%, and 2.19% in pediatric cases, respectively.

**Table 2:** pCNVs detected in prenatal and pediatric settings.

Type of pCNVs	Prenatal			Pediatric		
	No. Abn Cases	ADR	RF	No. Abn Cases	ADR	RF
**Genomic Disorders**						
**22q11.21**	**7**	**0.70**	**26.92**	**57**	1.74*	**17.82**
Del/Dup	4/3	0.40/0.30	15.38/11.54	46/11	1.40/0.34*	14.38/3.44
**15q11-15q13**	**1**	**0.10**	**3.85**	**37**	1.13*	**11.57**
Del/Dup	-/1	-/0.10	-/3.85	31/6	0.95/0.18	9.69/1.88
**16p11.2**				**22**	**0.67**	**6.88**
Del/Dup				15/7	0.46/0.21	4.69/2.19
**Xp22.31**				**17**	**0.52**	**5.32**
Del/Dup				7/10	0.21/0.31	2.19/3.13
**1q21**				**15**	**0.46**	**4.70**
Del/Dup				3/10	0.09/0.31	0.94/3.13
Del & Dup				2	0.06	0.63
**7q11.23**				**14**	**0.42**	**4.38**
Del/Dup				8/6	0.24/0.18	2.50/1.88
**16p13.11**	**3**	**0.30**	**11.54**	**10**	**0.30**	**3.13**
Del/Dup	3/-	0.30/-	11.54/-	4/6	0.12/0.18	1.25/1.88
**17q12**				**9**	**0.27**	**2.81**
Del/Dup				5/4	0.15/0.12	1.56/1.25
**3q29**				**7**	**0.21**	**2.19**
Del/Dup				6/1	0.18/0.03	1.88/0.31
**17p12**	**1**	**0.10**	**3.85**	**7**	**0.21**	**2.19**
Del/Dup	1/-	0.10/-	3.85/-	2/5	0.06/0.15	0.63/1.56
**17p13.3**	**2**	**0.20**	**7.69**	**6**	**0.18**	**1.88**
Del/Dup	1/1	0.10/0.10	3.85/3.85	4/2	0.12/0.06	1.25/0.63
**8p23.1**				**5**	**0.15**	**1.56**
Del/Dup				4/1	0.12/0.03	1.25/0.31
**5q35 deletion**				**5**	**0.15**	**1.56**
**15q13**	**2**	**0.20**	**7.69**	**4**	**0.12**	**1.25**
Del/Dup	1/1	0.10/0.10	3.85/3.85	1/3	0.03/0.09	0.31/0.94
**2q13**				**3**	**0.09**	**0.93**
Del/Dup				1/1	0.03/0.03	0.31/0.31
Del & Dup				1	0.03	0.31
**17p11.2**				**3**	**0.09**	**0.94**
Del/Dup				2/1	0.06/0.03	0.63/0.31
**7q35-7q36 deletion**				**3**	**0.09**	**0.94**
**17q21.31**				**2**	**0.06**	**0.62**
Del/Dup				1/1	0.03/0.03	0.31/0.31
**17q11.2 deletion**				**2**	**0.06**	**0.62**
**22q13.33 deletion**				**2**	**0.06**	**0.62**
**4q35 deletion**				**1**	**0.03**	**0.31**
**Xq28 duplication**	**1**	**0.10**	**3.85**	**1**	**0.03**	**0.31**
**Total**	**17**	**1.69**	**65.38**	**232**	7.08*	**72.50**
**Sporadic pCNVs**						
Subtelomeric pCNVs	5	0.50	19.23	35	1.07	10.94
Interstitial pCNVs	4	0.40	15.38	53	1.62*	16.56
**Total**	**9**	**0.90**	**36.42**	**88**	2.69*	**27.50**
**All pCNVs**	**26**	**2.59**	**100.00**	**320**	9.77*	**100.00**

pCNVs, pathogenic copy number variants; Del/Dup, deletion/duplication; ADR (abnormality detection rate by %): no. abn cases/1004 prenatal cases, no. abn cases/3276 pediatric cases (3276 pediatric cases including 3210 cases analyzed by aCGH and 66 cases by FISH only for specific microdeletions or microduplications). RF (relative frequency by%): no. abn cases/26 prenatal abn cases; no. abn case/320 pediatric abn cases. *Statistically different from ADR in prenatal setting (p < 0.05).

**Figure 4:**
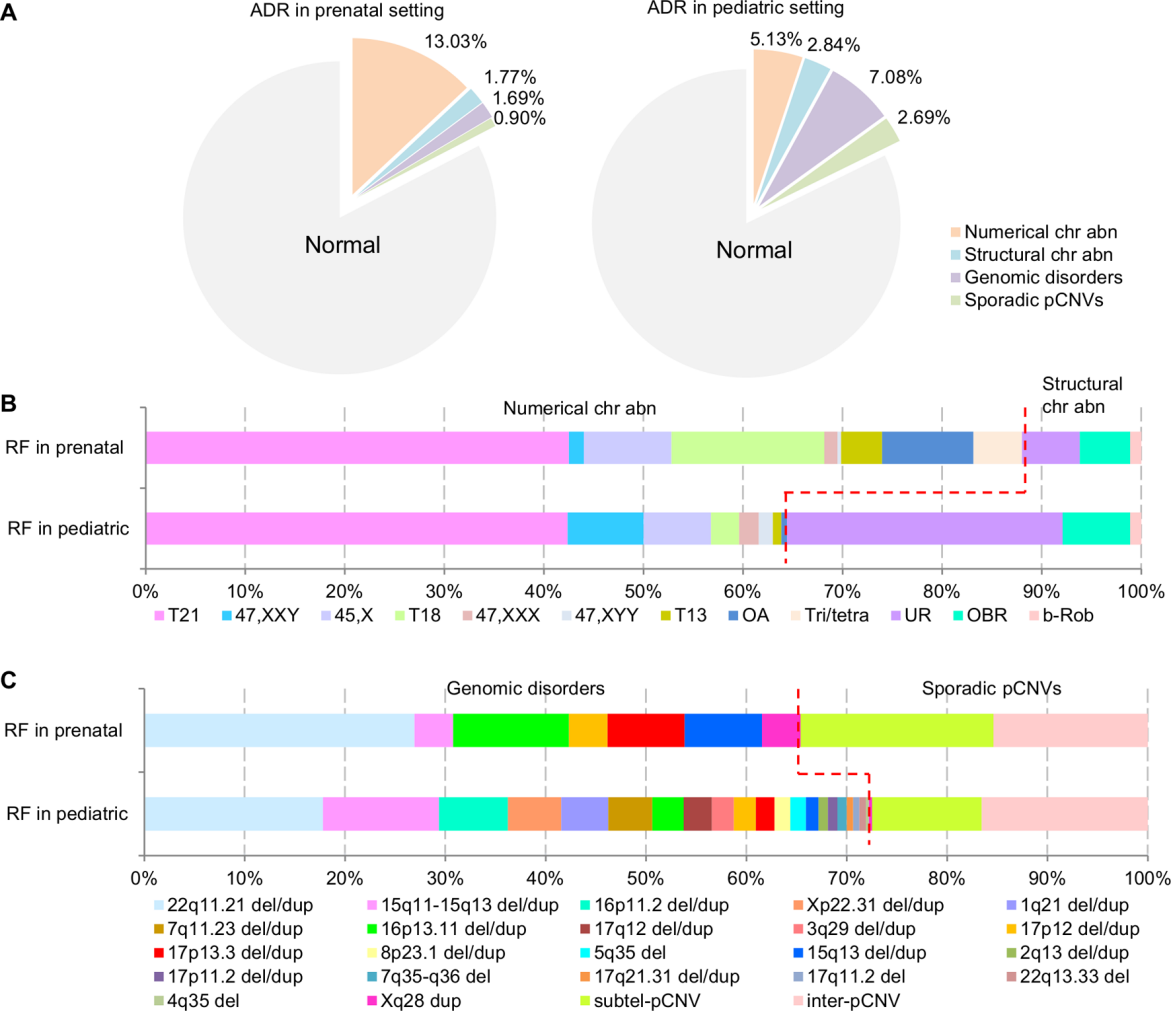
The ADR and RF of cytogenomic abnormalities detected in prenatal and pediatric settings. **(A)** The composite ADR of cytogenomic abnormalities in prenatal and pediatric settings. **(B/C)** The RF for the spectrum of chromosomal abnormalities and the spectrum of pCNVs in prenatal and pediatric settings. The red dot line separates the abnormalities into two groups per classification. ADR, abnormality detection rate; b-Rob, balanced Robertsonian translocations; chr abn, chromosomal abnormality; del/dup, deletion/duplication; inter-pCNV, interstitial pCNV; OA, other aneuploidy; OBR, other balanced rearrangements; pCNVs, pathogenic copy number variants; RF, relative frequency; subtel-pCNV, subtelomeric pCNV; T21, trisomy 21; T18, trisomy 18; T13, trisomy 13; Tri/tetra, triploid and tetraploid; UR, unbalanced rearrangements.

The composite ADR and RF for the spectrum of chromosomal abnormalities and pCNVs in prenatal and pediatric cases are shown in **Figure 4**. A comparison between the spectrum of chromosomal abnormalities and pCNVs showed a significant under detection of unbalanced rearrangements and pCNVs in current prenatal setting (p < 0.05).

### Correlating ADR With Disease Prevalence for Cytogenomic Abnormalities

**Figure 5:**
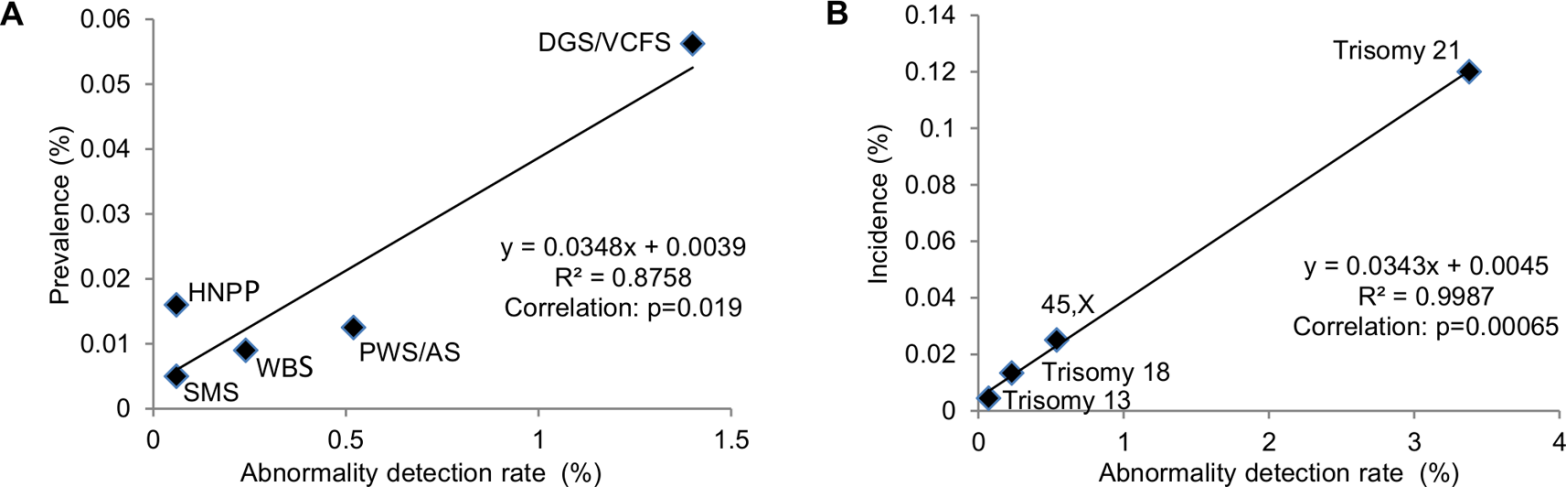
Correlation of prevalence with abnormal detection rate in pediatric setting by linear regression analysis. **(A)** Abnormality detection rate in pediatric setting was plotted against the reported prevalence for DGS/VCFS (velocardiofacial syndrome), WBS (Williams-Beuren syndrome), PWS/AS (Prader-Willi/Angelman syndromes), SMS (Smith-Magenis syndrome), and HNPP (hereditary neuropathy with liability to pressure palsies). **(B)** Abnormality detection rate in pediatric setting was plotted against the reported incidences for common aneuploidies trisomy 21, 45,X, trisomy 18, and trisomy 13.

Correlating ADR from current diagnostic practice with known prevalence of common genomic disorders was used to extrapolate the prevalence of rare genomic disorders (Gillentine et al., 2018). The correlation between the reported prevalence of selected genomic disorders with their ADR at Yale Clinical Cytogenetics Laboratory was determined (Y = 0.0348X + 0.0039; R² = 0.8758, p = 0.019) (**Figure 5A** and **Table 3**). Using this linear regression correlation, the prevalence of other syndromic genomic disorders was estimated as the following: 0.009% (1/10965) for 1q21.1 deletion syndrome, 0.017% (1/5960) for 1q21.1 duplication syndrome, 0.010% (1/9839) for 3q29 deletion syndrome, 0.020% (1/5025) for 16p11.2 deletion syndrome, and 0.009% (1/10965) for 17q12 deletion syndrome. The prevalence of detected recurrent genomic disorders and the total pCNVs was estimated to be 0.250% (1/396) and 0.344% (1/291), respectively. The estimated prevalence of these genomic disorders was in range with that from another report (Gillentine et al., 2018). This result also indicated that the aCGH was effective in detecting common genomic disorders in current pediatric setting.

**Table 3:** Known and estimated prevalence of pCNV by linear regression analysis.

Type of abnormality	Reported prevalence*	Estimated prevalence
DiGeorge/velocardiofacial syndrome	0.0625%–0.05% (1/1,600–1/2,000)	/
Williams-Beuren syndrome	0.013%–0.005% (1/7,500–1/20,000)	/
Prader-Willi/Angelman syndrome	0.014%–0.011% (1/7,142–1/9,090)	/
Smith-Magenis syndrom	0.006%%–0.004% (1/15,000–1/25,000)	/
Hereditary neuropathy with liability to pressure palsies	0.016% (1/6250)	/
1q21.1 deletion syndrome	/	0.009% (1/10,965)
1q21.1 duplication syndrome	/	0.017% (1/5,960)
3q29 deletion syndrome	/	0.010% (1/9,839)
16p11.2 deletion syndrome	/	0.020% (1/5,025)
17q12 deletion syndrome	/	0.009% (1/10,965)
**Recurrent genomic disorders**	**/**	**0.250% (1/396)**
**All pCNVs**	**/**	**0.344% (1/291)**

*Reported prevalence from Greenberg et al., 1991; Kyllerman et al., 1995; Meretoja et al., 1997; Strømme et al., 2002; Oiglane-Shlik et al., 2006; and Shprintzen 2008.

The correlation between the incidences of selected chromosomal abnormalities in live births with their ADR in pediatric patients was determined (Y = 0.0343X + 0.0045; R² = 0.9987, p = 0.00065) (**Figure 5B** and **Table 4**). Using this linear regression correlation, the incidence of other chromosomal abnormalities was estimated as: 0.0254% (1/3933) for 47,XXY, 0.01% (1/10012) for 47,XXX, 0.0083% (1/12088) for 47,XYY, and 0.0076% (1/13180) for balanced Robertsonian translocations. These estimated incidences were much lower than the previously published incidences in live births (Nussbaum et al., 2007). This result indicated that current pediatric genetic evaluation was effective in identifying trisomy 21, 45,X, trisomy 18, and trisomy 13, but was not effective in identifying other sex chromosomal aneuploidies and balanced Robertsonian translocations.

**Table 4:** Estimated incidence of chromosomal abnormalities by linear regression analysis.

Type of abnormality	Incidence in live births*	Under-estimated incidence
45,X	0.025% (1/4,000)	/
trisomy 21	0.12% (1/830)	/
trisomy 18	0.0133% (1/7,500)	/
trisomy 13	0.0044% (1/22,700)	/
47,XXY	0.1% (1/1,000)	0.0254% (1/3,933)
47,XYY	0.1% (1/1,000)	0.0083% (1/12,088)
47,XXX	0.11% (1/900)	0.0100% (1/10,012)
Balanced Robertsonian	0.09% (1/1,100)	0.0076% (1/13,180)

*Incidence of chromosome abnormalities by Nussbaum et al., 2007.

### Estimation of Diagnostic Efficacy From Served Population

The Yale Clinical Cytogenetics Laboratory provides diagnostic services to the population in New Haven and its surrounding counties in southern Connecticut. The population in New Haven County is 0.9 million and in Connecticut State is 3.6 million. Based on the 1.0% birth rate in annual registration reports (2009 to 2015) from Connecticut Department of Public Health, there should be about 36,000 newborns in Connecticut per year. With known incidence of Down syndrome (DS) of 1/830 and DGS of 1/4,000, 45 newborns with DS and 9 newborns with DGS are expected in the State of Connecticut each year. This study detected 377 DS cases and 50 DGS cases in a 10-year interval, with an estimated diagnosis of 38 DS cases and 5 DGS cases per year accounting for 55%–85% of the expected cases in Connecticut. Considering there were overlapping DS cases from prenatal testing and follow up pediatric testing, it was estimated that the patients referred for cytogenomic testing were likely from a base of 60% of the Connecticut population, which is about two million residing in New Haven County and the surrounding area. Two million was used as the population size our lab served.

To estimate the diagnostic efficacy, the number of cases of chromosomal abnormalities and pCNVs based on their newborn incidences was compared with diagnostic results based on one million population size (**Table 5**). It could be assumed that diagnostic efficacy at 90% or higher is highly effective, 50%–90% is variably effective, and below 50% is under detection. The overall diagnostic efficacy for cytogenomic abnormalities was about 29% in prenatal cases and 35% in pediatric cases. For chromosome abnormalities, the diagnostic efficacy of 92% for prenatal detection of DS and TS (45,X) should be considered highly effective. The efficacy for detecting 47,XXY, 47,XXX, 47,XYY, and Robertsonian translocations ranged from 1%–4% in prenatal practice and 2%–14% in pediatric evaluation indicated under detection. Assumed no overlapping cases between prenatal and pediatric cases, 42% of chromosomal abnormalities and 4% of pCNVs were detected prenatally and an additional 28% chromosomal abnormalities and 48% of pCNVs were detected postnatally.

**Table 5:** Estimated diagnostic efficacy for chromosomal abnormalities and pCNVs.

Type of cytogenomic abnormality	Incidence in newborns^a^	Expected cases per 10,000 newborns^b^	Prenatal setting		Pediatric setting	
			Detected cases per 10,000 newborns^b^	Estimated diagnostic efficacy	Detected cases per 10,000 newborns^b^	Estimated diagnostic efficacy
Trisomy 21	1/830	12	11	92%	7.5	63%
45,X	1/4,000	2.5	2.3	92%	1.2	48%
47,XXY	1/1,000	10	0.4	4%	1.4	14%
47,XXX	1/900	11	0.4	4%	0.4	4%
47,XYY	1/1,000	10	0.1	1%	0.3	3%
Balanced Rob.	1/1,100	9	0.3	3%	0.2	2%
**All chromosome abnormalities**	**1/154**	**64**	**27**	**42%**	**18**	**28%**
DGS/VCFS	1/4,000	2.5	0.2	8%	2.3	92%
16p11.2 del	1/5,800	1.7	0		0.75	44%
1q21.1 del	1/7,400	1.4	0		0.25	18%
PWS/AS	1/8,000	1.25	0		0.85	68%
WBS	1/10,000	1	0		0.4	40%
**All recurrent genomic disorders**	**1/396**	**25**	**0.85**	**3%**	**11.6**	**46%**
**All pCNVs**	**1/291**	**33**	**1.3**	**4%**	**16**	**48%**
**Total cytogenomic abnormalities**	**1/100**	**97**	**28.3**	**29%**	**34**	**35%**

Rob, Robertsonian translocation; DGS/VCFS, DiGeorge/velocardiofacial syndrome; PWS/AS, Prader-Willi/Angelman syndrome; WBS, Williams-Beuren syndrome; pCNVs, pathogenic copy number variants.

Incidence of cytogenomic abnormalities in newborns by Nussbaum et al., 2007, Wei et al., 2013 and this study;

Expected cases derived from incidence and detected cases from average annual abnormal cases based on 1.0% birth rate from Connecticut Department of Public Health and estimated 2 million population served by Yale Clinical Cytogenetics Laboratory. Diagnostic efficacy was estimated by detected cases/expected cases.

The ratio of DS cases vs DGS cases (DS/DGS) from their incidences should be about 4 to 5 (1/830 vs 1/4,000). This DS/DGS ratio could be used as a simple measurement for efficacy in detecting chromosome abnormalities and pCNVs. The DS/DGS ratio from this study was 57 (227/4) in prenatal cases and 3.3 (150/46) in pediatric cases. The extremely high prenatal DS/DGS ratio indicated under detection of DGS in the prenatal testing. The lower pediatric DS/DGS ratio may be affected by decreased cases of DS from effective prenatal testing.

## Discussion

It has been over 10 years since the integration of microarray analysis into the clinical cytogenetics diagnostic services for prenatal and pediatric cases. The dual processing of DNA-based aCGH and cell-based karyotyping and FISH ensured analytical validity and diagnostic accuracy for detecting cytogenomic abnormalities. The accuracy from aCGH analysis can achieve over 99% sensitivity and specificity (Xiang et al., 2008). This retrospective analysis was based on consecutive clinical referrals and standardized aCGH, karyotyping, and FISH analyses performed in a laboratory, a similar spectrum of cytogenomic abnormalities was observed but different ADR and RF for types of abnormalities were obvious between prenatal and pediatric cases.

### Difference of Diagnostic Yield in the Spectrum of Cytogenomic Abnormalities Between the Prenatal and Pediatric Settings

In prenatal cases, chromosomal abnormalities and pCNVs accounted for 95% (534/560) and 5% (26/560) of the total abnormalities, respectively. While in pediatric patients, chromosomal abnormalities and pCNVs accounted for 53% (354/674) and 47% (320/674) of the total abnormalities, respectively. Among cases with chromosomal abnormalities, numerical and structural chromosomal abnormalities accounted for 88% and 12% in prenatal cases, and 64% and 36% of pediatric patients, respectively. Under detection of 47,XXY, 47,XXX, 47,XYY, and balanced Robertsonian translocations was evident from their ADR and RF, as well as under estimated incidences of these abnormalities in the liner regression analysis. The mild phenotypes of these cases during childhood likely explain the under detection. Clinical attention in a different setting or at a different time such as genetic evaluation for reproductive failure or other age-related symptoms may allow detection of some portion of these chromosomal abnormalities later.

The diagnostic efficacy for pCNVs showed a clear under detection in prenatal practice and variable efficiency in pediatric practice. It is estimated that only 8% of DGS was detected in prenatal diagnosis. In pediatric genetic analysis, the diagnostic efficacy of 92% for DGS was highly effective and of 40%–68% for WBS, PWS/AS and 16p11.2 deletion showed variability. For recurrent genomic disorders and all pCNVs, approximately 3% and 4% were detected prenatally and 46% and 48% were detected postnatally, respectively. From the linear regression analysis, the prevalence of other common genomic disorders such as 1q21.1 microdeletion and microduplication syndrome, 16p11.2 microdeletion syndrome, 3q29 deletion syndrome, and 17q12 deletion syndrome was similar to those reported from other studies (Stefansson et al., 2014; Gillentine et al., 2018). The prevalence of all recurrent genomic disorders was estimated to be 1/396 and of all pCNVs was 1/291. Combining the prevalence of 1/291 for pCNVs with 1/154 for chromosomal abnormalities (Nussbaum et al., 2007), the prevalence of all cytogenomic abnormalities is about 1/100.

Current phenotype-first analysis could lead to under detection of recurrent genomic disorders and sporadic pCNVs due to variable expressivity and incomplete penetrance causing under estimation of prevalence. A genotype-first epidemiological study in a large population is the best way to determine the prevalence of pCNVs. Although the estimated prevalence for recurrent genomic disorders or pCNVs varied and thus affect the evaluation of diagnostic efficacy, approaches to improve the diagnostic yield of pCNVs should be considered.

### Approaches to Improve the Diagnostic Yield of Cytogenomic Abnormalities in Prenatal Clinical Practice

Prenatal diagnosis is currently performed on pregnant women at risk for genetic disorders. The clinical indications for cytogenomic analysis include abnormal results by non-invasive prenatal testing (NIPT) of maternal plasma cell-free fetal DNA, abnormal ultrasound findings (aUS), abnormal maternal serum screening (aMSS), advanced maternal age (AMA), family history (FH) of chromosomal abnormalities, parental anxiety, and other adverse fetal health events. Since 2000, the development of sensitive ultrasonic technology and utilization of maternal serum markers have increased aUS and aMSS as indications for prenatal cytogenetic analysis and decreased AMA as an indication (Li et al., 2011; Meng et al., 2015). The introduction of NIPT has significantly reduced the invasive AF and CVS procedures and increased the ADR; which explained the continuous decrease of prenatal cases during 2008–2012 in this report. A recent study showed that the NIPT performed significantly better in predicting sex chromosome trisomies than monosomy X; which could potentially improve the under detection of sex chromosome aneuploidy in prenatal diagnosis (Xu et al., 2019). The integration of aCGH in prenatal diagnosis has extended its diagnostic scope to include pCNVs (Li et al., 2016; Chai et al., 2019). In this study, aCGH was performed in about one third of total prenatal cases with an ADR of 2.59% for pCNVs. Application of aCGH to the other two third of the cases with normal karyotype is expected to detect a similar percentage of pCNVs. A multicenter study by the National Institute of Child Health and Human Development (NICHD) showed that pCNVs were detected in 2.5% of all prenatal cases by microarray analysis (Wapner et al., 2012). This NICHD multicenter study revealed that in samples with a normal karyotype, pCNVs were detected in 6.0% of fetuses with ultrasound anomalies and in 1.7% of those with AMA, aMSS or parental anxiety. A study on 5,026 consecutive prenatal specimens by high resolution microarray analysis detected pCNV in 3% of cases (Wang et al., 2019). A systematic review of 19 studies and meta-analysis of the 10 largest studies in a pooled cohort of 10,614 fetuses showed that 0.84% of fetuses investigated by invasive prenatal testing due to AMA and parental anxiety had a pathogenic clinically signiﬁcant submicroscopic aberrations; of which, pCNVs associated with early-onset syndromic disorders accounted for 0.37% of cases, pCNVs associated with late-onset syndromic disorders accounted for 0.11% of cases, and susceptible CNVs in 0.30% of cases (Srebniak et al., 2018). A cohort study found an excess ADR of 4.1% by microarray analysis over conventional karyotyping when the clinical indication for testing was abnormal fetal ultrasound findings; and this excess detection rate increased to 10% in the author’s meta-analysis (Hillman et al., 2013). In another study of 1,033 fetuses with ultrasound anomalies, pathogenic submicroscopic abnormalities were identified in 5.5% of cases (Srebniak et al., 2016). Lack of easily detectable anatomical abnormality in many of the pCNVs likely contribute to their low detection rate in the prenatal setting. Several studies reported that ultrasonographic fetal anomalies such as heart defects, overgrowth or undergrowth, and limb defects had been used as clinical indications for prenatal testing of DGS, Jacobsen syndrome, Cri du Chat syndromes, split hand/foot malformations and Simpson-Galobi-Bemhel syndrome (Cook et al., 2014; Xu et al., 2014b; Meng et al., 2015; DiMaio et al., 2017).

Currently, prenatal chromosomal microarray analysis is recommended for pregnant women with a fetus showing one or more major structural abnormalities identified by ultrasonographic examination (Committee on Genetics and the Society for Maternal-Fetal Medicine, 2016). The major challenge for prenatal diagnosis is to improve the screening efficiency and thus increase the diagnostic yield of pCNVs for better management of birth defects, especially for the well-recognized syndromic genomic disorders (Martin et al., 2018; Wang et al., 2019). However, most prenatal screening programs mainly determine the risk of common aneuploidies and seldom predict the risk of submicroscopic pCNVs (Kazerouni et al., 2011; Alamillo et al., 2013; Benn et al., 2013). Improved SNP-based NIPT was developed to screen for a subset of submicroscopic abnormalities. A study of 21,948 samples submitted for screening of DGS/VCFS by SNP-based NIPT identified 95 cases as high risk for fetal DGS/VCFS; 61 of those were further analyzed by a diagnostic test and confirmed 11 true positives and 50 false positives, indicating a positive predictive value of 18.0% (Gross et al., 2016). The performance of SNP-based NIPT in 80,449 referrals for DGS/VCFS and 42,326 referrals for 1p36, cri-du-chat, Prader-Willi/Angelman microdeletion syndromes and a comparison of original screening protocol with a revision that reflexively sequenced high-risk calls at a higher depth of reads were retrospectively analyzed (Martin et al., 2018). The positive predictive value of the original screening was 15.7% for DGS/VCFS and 5.2% for the other four disorders combined. With the revised protocol, these values increased to 44.2% for DGS/VCFS and 31.7% for the others. The risk of pCNVs under different prenatal clinical indications is summarized in **Table 6**.

In summary, to improve the yield of pCNVs in prenatal diagnosis, the first approach is to extend aCGH for more prenatal cases; the second approach is to establish reliable correlations between genomic disorders and ultrasonagraphic fetal anomalies and use them as clinical indications in genetic counseling for a complete prenatal cytogenomic testing; and the third approach is to enhance the analytical resolution of NIPT to screen for both chromosomal aneuploidies and common genomic disorders. The enhanced NIPT results could be a more effective predicator for pCNVs and should be considered for the general pregnant population regardless of maternal age and other clinical indications (Wapner et al., 2015).

**Table 6:** Clinical indications and the risk of pCNVs from prenatal studies.

Indications	Risk of pCNVs	References
Mix indications	2.5%–3%	Wapner et al., 2012, Wang et al., 2019, this study
AMA, aMSS, parental anxiety	0.84%–1.7%	Wapner et al., 2012, Srebniak et al., 2018
Abnormal ultrasound findings	4.1%–10%	Wapner et al., 2012, Hillman et al., 2013, Srebniak et al., 2016
NIPT^a^	5.2%–18%	Gross et al., 2016, Martin et al., 2018
NIPT enhanced ^b^	31.7%–44.2%	Martin et al., 2018

AMA, advanced maternal age; aMSS, abnormal maternal serum screening; NIPT, non-invasive prenatal testing; pCNVs, pathogenic copy number variants;

^a^in screening for 22q11.2 deletion syndrome and 1p36, cri-du-chat, Prader-Willi, and Angelman microdeletion syndromes.

^b^enhanced NIPT by using high depth sequencing.

### Cytogenomic Aberrations Not Detectable by Microarray Analysis

In this study, the ADR for balanced structural chromosomal abnormalities in prenatal and pediatric cases was 0.92% and 0.63%, respectively, which were consistent with the 0.78%–1.3% reported in the ACMG practice resource (Waggoner et al., 2018). These balanced structural chromosomal abnormalities, including reciprocal translocations, insertions, inversions, and balanced Robertsonian translocations, are not detectable by microarray analysis. Professional recommendations stated that ordering providers should be aware of cytogenomic aberrations not detectable by microarray analysis, including those relevant to various microarray platforms (e.g., SNP versus oligonucleotide) (Manning and Hudgins, 2010). Although most of balanced rearrangements are benign, several studies indicated that balanced structural rearrangements associated with direct functional gene disruption, truncation, or disruption of regulatory domains would contribute to pathogenic phenotype (Brownstein et al., 2008; Ordulu et al., 2016; Zepeda-Mendoza et al., 2017). Therefore, conventional karyotyping remains valuable in the detection of balanced structural chromosomal abnormalities and mosaicism.

## Conclusion

The diagnostic yield of cytogenomic abnormalities was evaluated in current prenatal and pediatric settings over a period of 10 years. Detailed analysis on the spectrum of cytogenomic abnormalities indicated high efficacy in prenatal detection of DS and TS and postnatal detection of DGS. Under detection of pCNVs in the prenatal setting and of other sex chromosome numerical abnormalities in both prenatal and postnatal settings were noted. Efficacy for detecting other pCNVs in pediatric setting was variable. Expansion of aCGH analysis to more prenatal cases, more reliable correlations between genomic disorders and fetal ultrasonographic anomalies, and enhanced NIPT screening for well-recognized syndromic genomic disorders are approaches that could improve diagnostic yield of cytogenomic abnormalities in prenatal cases. Better understanding of clinical presentations for sex chromosome numerical abnormalities and pCNVs could help to improve diagnostic yield for pediatric cases. The prevalence of cytogenomic abnormalities in about 1% of newborns may call for the change from phenotype-first detection to genotype-first *surveillance* in clinical practice and potentially contribute to a better clinical management of cytogenomic abnormalities in the future.

## Data Availability Statement

All datasets generated for this study are included in the article/**Supplementary Material**.

## Author Contributions

PL and HZ conceived and directed the study. MM, AB, JM, MS-M, and HZ referred patients and reviewed the clinical data. HC, AD, BG, FX, and QZ performed the cytogenomic analyses and PL and JW interpreted the results. HC conducted the data analysis and the statistical work. HC, PL, HZ, and MM wrote, edited, and revised the manuscript. All authors read and approved the submitted version.

## Funding

QZ was partly supported by the National Natural Science Foundation of China (81601299).

## Conflict of Interest

FX is currently employed by company Prevention Genetics. The remaining authors declare that the research was conducted in the absence of any commercial or ﬁnancial relationships that could be construed as a potential conﬂict of interest.

## References

[B1] ACOG Committee Opinion No. 446 (2009). array comparative genomic hybridization in prenatal diagnosis. Obstet. Gynecol. 114, 1161–1163. 10.1097/AOG.0b013e3181c33cad 20168129

[B2] Ahn J. W. Bint S. Bergbaum A. Mann K. Hall R. P. Ogilvie C. M. (2013). Array CGH as a first line diagnostic test in place of karyotyping for postnatal referrals - results from four years’ clinical application for over 8,700 patients. Mol. Cytogenet. 6, 16. 10.1186/1755-8166-6-16 23560982 PMC3632487

[B3] Alamillo C. M. Krantz D. Evans M. Fiddler M. Pergament E. (2013). Nearly a third of abnormalities found after first-trimester screening are different than expected: 10- year experience from a single center. Prenat. Diagn. 33, 251–256. 10.1002/pd.4054 23354915

[B4] Benn P. Cuckle H. Pergament E. (2013). Non-invasive prenatal testing for aneuploidy: current status and future prospects. Ultrasound Obstet. Gynecol. 42, 15–33. 10.1002/uog.12513 23765643

[B5] Brownstein C. A. Adler F. Nelson-Williams C. Iijima J. Li P. Imura A. (2008). A translocation causing increased alpha-klotho level results in hypophosphatemic rickets and hyperparathyroidism. Proc. Natl. Acad. Sci. U. S. A. 105, 3455–3460. 10.1073/pnas.0712361105 18308935 PMC2265125

[B6] Chai H. DiAdamo A. Grommisch B. Boyle J. Amato K. Wang D. (2019). Integrated FISH, karyotyping and aCGH analyses for effective prenatal diagnosis of common aneuploidies and other cytogenomic abnormalities. Med. Sci. 7, 16. 10.3390/medsci7020016 PMC6410168 30678103

[B7] Committee on Genetics and the Society for Maternal-Fetal Medicine (2016). Committee opinion No.682: Microarrays and next-generation sequencing technology: The use of advanced genetic diagnostic tools in obstetrics and gynecology. Obstet. Gynecol. 128, e262–e268. 10.1097/AOG.0000000000001817 27875474

[B8] Cook S. Wilcox K. Grommisch B. Li P. Xu F. (2014). Prenatal diagnosis of Xq26.1-q26.3 duplication in two fetuses of a woman with gonadal mosaicism. N. Am. J. Med. Sci. 7, 176–179. 10.7156/najms.2014.0704176

[B9] DiMaio S. M. Yang H. Mahoney M. McGrath J. Li P. (2017). Familial *GPC3* and *GPC4-TFDP3* deletions at Xq26 associated with Simpson-Golabi-Behmel syndrome. Meta Gene 11, 147–151. 10.1016/j.mgene.2016.08.008

[B10] Gillentine M. A. Lupo P. J. Stankiewicz P. Schaaf C. P. (2018). An estimation of the prevalence of genomic disorders using chromosomal microarray data. J. Hum. Genet. 63, 795–801. 10.1038/s10038-018-0451-x 29691480 PMC6019170

[B11] Greenberg F. Guzzetta V. Montes de Oca-Luna R. Magenis R. E. Smith A. C. Richter S. F. (1991). Molecular analysis of the Smith-Magenis syndrome: a possible contiguous-gene syndrome associated with del(17)(p11.2). Am. J. Hum. Genet. 49, 1207–1218. 1746552 PMC1686451

[B12] Gross S. J. Stosic M. McDonald-McGinn D. M. Bassett A. S. Norvez A. Dhamankar R. (2016). Clinical experience with single-nucleotide polymorphism-based non-invasive prenatal screening for 22q11.2 deletion syndrome. Ultrasound Obstet. Gynecol. 47, 177–183. 10.1002/uog.15754 26396068 PMC5064640

[B13] Hillman S. C. McMullan D. J. Hall G. Togneri F. S. James N. Maher E. J. (2013). Use of prenatal microarray: prospective cohort study and systematic review and meta-analysis. Ultrasound Obstet. Gynecol. 41, 610–620. 10.1002/uog.12464 23512800

[B14] Kazerouni N. N. Currier R. J. Flessel M. Goldman S. Hennigan C. Hodgkinson C. (2011). Detection rate of quadruple-maker screening determined by clinical follow-up and registry datain the statewide California program, July 2007 to February 2009. Prenat. Diagn. 31, 901–906. 10.1002/pd.2802 21706514

[B15] Kearney H. M. Thorland E. C. Brown K. K. Quintero-Rivera F. South S. T.Working Group of the American College of Medical Genetics Laboratory Quality Assurance Committee (2011). American College of Medical Genetics standards and guidelines for interpretation and reporting of postnatal constitutional copy number variants. Genet. Med. 13, 680–685. 10.1097/GIM.0b013e3182217a3a 21681106

[B16] Kyllerman M. (1995). On the prevalence of Angelman syndrome. Am. J. Med. Genet. 59, 405. 10.1002/ajmg.1320590331 8599374

[B17] Li P. Pomianowski P. DiMaio M. S. Florio J. R. Rossi M. R. Xiang B. (2011). Genomic characterization of prenatally detected chromosomal structural abnormalities using oligonucleotide array comparative genomic hybridization. Am. J. Med. Genet. 155A, 1605–1615. 10.1002/ajmg.a.34043 21671377 PMC3745591

[B18] Li P. DiAdamo A. Grommisch B. Boyle J. Amato K. Wang D. (2016). Diagnostic yield of cytogenomic abnormalities in current prenatal diagnosis: a retrospective analysis in a clinical cytogenetics laboratory. N. Am. J. Med. Sci. 9, 136–140. 10.7156/najms.2016.0904136

[B19] Manning M. Hudgins L. Professional Practice and Guidelines Committee (2010). Array-based technology and recommendations for utilization in medical genetics practice for detection of chromosomal abnormalities. Genet. Med. 12, 742–745. 10.1097/GIM.0b013e3181f8baad 20962661 PMC3111046

[B20] Martin K. Iyengar S. Kalyan A. Lan C. Simon A. L. Stosic M. (2018). Clinical experience with a single-nucleotide polymorphism-based non-invasive prenatal test for five clinically significant microdeletions. Clin. Genet. 93, 293–300. 10.1111/cge.13098 28696552

[B21] Meng J. Matarese C. Crivello J. Wilcox K. Wang D. DiAdamo A. (2015). Changes in and efficacies od indications for invasive prenatal diagnosis of cytogenomic abnormalities: 13 years of experience in a single center. Med. Sci. Monit. 21, 1942–1948. 10.12659/MSM.893870 26143093 PMC4497468

[B22] Meretoja P. Silander K. Kalimo H. Aula P. Meretoja A. Savontaus M. L. (1997). Epidemiology of hereditary neuropathy with liability to pressure palsies (HNPP) in south western Finland. Neuromuscul. Disord. 7, 529–532. 10.1016/S0960-8966(97)00100-4 9447611

[B23] Miller. D. T. Adam M. P. Aradhya S. Biesecker L. G. Brothman A. R. Carter N. P. (2010). Consensus statement: chromosomal microarray is a first-tier clinical diagnostic test for individuals with developmental disabilities or congenital anomalies. Am. J. Hum. Genet. 86, 749–764. 10.1016/j.ajhg.2010.04.006 20466091 PMC2869000

[B24] Ning Y. Roschke A. Smith A. C. Macha M. Precht K. Riethman H. (1996). A complete set of human telomeric probes and their clinical application. Nat. Genet. 14, 86–89. 10.1038/ng0996-86 8782825

[B25] Nussbaum R. L. McInnes R. R. Willard H. F. Hamosh A. (2007). Thompson & Thompson Genetics in Medicine. Philadelphia, PA: Saunders Elsevier Publishers, 76.

[B26] Ordulu Z. Kammin T. Brand H. Pillalamarri V. Redin C. E. Collins R. L. (2016). Structural chromosomal rearrangements require nucleotide- level resolution: Lessons from next-generation sequencing in prenatal diagnosis. Am. J. Hum. Genet. 99, 1015–1033. 10.1016/j.ajhg.2016.08.022 27745839 PMC5097935

[B27] Oiglane-Shlik E. Talvik T. Zordania R. Põder H. Kahre T. Raukas E. (2006). Prevalence of Angelman syndrome and Prader-Willi syndrome in Estonian children: sister syndromes not equally represented. Am. J. Med. Genet. 140A, 1936–1943. 10.1002/ajmg.a.31423 16906556

[B28] Park S. J. Jung E. H. Ryu R. S. Kang H. W. Chung H. D. Kang H. Y. (2013). The clinical application of array CGH for the detection of chromosomal defects in 20,126 unselected newborns. Mol. Cytogenet. 6, 21. 10.1186/1755-8166-6-21 23725218 PMC3682880

[B29] Ried T. Landes G. Dackowski W. Klinger K. Ward D. C. (1992). Multicolor fluorescence *in situ* hybridization for the simultaneous detection of probe sets for chromosomes 13, 18, 21, X and Y in uncultured amniotic fluid cells. Hum. Mol. Genet. 5, 307–313. 10.1093/hmg/1.5.307 1303206

[B30] Shaffer L. G. (2005). American College of Medical Genetics Professional Practice and Guidelines Committee, American College of Medical Genetics guideline on the cytogenetic evaluation of the individual with developmental delay or mental retardation. Genet. Med. 7, 650–654. 10.1097/01.gim.0000186545.83160.1e 16301868 PMC3110947

[B31] Shprintzen R. J. (2008). Velo-cardio-facial syndrome: 30 Years of study. Dev. Disabil. Res. Rev. 14, 3–10. 10.1002/ddrr.2 18636631 PMC2805186

[B32] Smeets D. F. C. M. (2004). Historical prospective of human cytogenetics: from microscope to microarray. Clin. Biochem. 37, 439–446. 10.1016/j.clinbiochem.2004.03.006 15183291

[B33] South S. T. Lee C. Lamb A. N. Higgins A. W. Kearney H. M.Working Group for the American College of Medical Genetics and Genomics Laboratory Quality Assurance Committee (2013). ACMG Standards and Guidelines for constitutional cytogenomic microarray analysis, including postnatal and prenatal applications: revision 2013. Genet. Med. 15, 901–909. 10.1038/gim.2013.129 24071793

[B34] Srebniak M. I. Diderich K. E. Joosten M. Govaerts L. C. Knijnenburg J. de Vries F. A. (2016). Prenatal SNP array testing in 1000 fetuses with ultrasound anomalies: causative, unexpected and susceptibility CNVs. Eu. J. Hum. Genet. 24, 645–651. 10.1038/ejhg.2015.193 PMC4930096 26328504

[B35] Srebniak M. I. Joosten M. Knapen M. F. C. M. Arends L. R. Polak M. van Veen S. (2018). Frequency of submicroscopic chromosomal aberrations in pregnancies without increased risk for structural chromosomal aberrations: systematic review and meta-analysis. Ultrasound Obstet. Gynecol. 51, 445–452. 10.1002/uog.17533 28556491

[B36] Stefansson H. Meyer-Lindenberg A. Steinberg S. Magnusdottir B. Morgen K. Arnarsdottir S. (2014). CNVs conferring risk of autism or schizophrenia affect cognition in controls. Nat. 50, 361–366. 10.1038/nature12818 24352232

[B37] Strømme P. Bjørnstad P. G. Ramstad K. (2002). Prevalence estimation of Williams syndrome. J. Child Neurol. 17, 269–271. 10.1177/088307380201700406 12088082

[B38] Veltman J. A. (2006). Genomic microarrays in clinical diagnosis. Curr. Opin. Pediatr. 18, 598–603. 10.1097/MOP.0b013e3280105417 17099357

[B39] Waggoner D. Wain K. E. Dubuc A. M. Conlin L. Hickey S. E. Lamb A. N. (2018). Yield of additional genetic testing after chromosomal microarray for diagnosis of neurodevelopmental disability and congenital anomalies: a clinical practice resource of the American College of Medical Genetics and Genomics (ACMG). Genet. Med. 20, 1105–1113. 10.1038/s41436-018-0040-6 29915380 PMC6410698

[B40] Wang J. C. Radcliff J. Coe S. J. Mahon L. W. (2019). Effects of platforms, size filter cutoffs, and targeted regions of cytogenomic microarray on detection of copy number variants and uniparental disomy in prenatal diagnosis: Results from 5026 pregnancies. Prenat. Diagn. 39, 137–156. 10.1002/pd.5375 30734327

[B41] Wapner R. J. Martin C. L. Levy B. Ballif B. C. Eng C. M. Zachary J. M. (2012). Chromosomal microarray versus karyotyping for prenatal diagnosis. N. Engl. J. Med. 367, 2175–2184. 10.1056/NEJMoa1203382 23215555 PMC3549418

[B42] Wapner R. J. Babiarz J. E. Levy B. Stosic M. Zimmermann B. Sigurjonsson S. (2015). Expanding the scope of noninvasive prenatal testing: detection of fetal microdeletion synfrome. Am. J. Obstet. Gynecol. 212, 332e1–332.e9. 10.1016/j.ajog.2014.11.041 25479548

[B43] Wei Y. Xu F. Li P. (2013). Technology-driven and evidence-based genomic analysis for integrated pediatric and prenatal genetic evaluation. J. Genet. Genomics 40, 1–14. 10.1016/j.jgg.2012.12.004 23357340

[B44] Xiang B. Hemingway S. Qumsiyeh M. Li P. (2006). CytoAccess: A relational laboratory information management system for a clinical cytogenetics laboratory. J. Assoc. Genet. Technol. 32, 168–170. 17130662

[B45] Xiang B. Li A. Valentin D. Novak N. Zhao H. Y. Li P. (2008). Analytical and clinical validity of whole genome oligonucleotide array comparative genomic hybridization for pediatric patients with mental retardation and developmental delay. Am. J. Med. Genet. 146A, 1942–1954. 10.1002/ajmg.a.32411 18627053

[B46] Xiang B. Zhu H. Shen Y. Miller D. T. Lu K. Hu X. (2010). Genome-wide oligonucleotide array comparative genomic hybridization for etiological diagnosis of mental retardation: a multicenter experience of 1499 clinical cases. J. Mol. Diagn. 12, 204–212. 10.2353/jmoldx.2010.090115 20093387 PMC2871727

[B47] Xu F. Li L. Schulz V. P. Gallagher P. G. Xiang B. Zhao H. (2014a). Cytogenomic mapping and bioinformatic mining reveal interacting brain expressed genes for intellectual disability. Mol. Cytogenet. 7, 4–15. 10.1186/1755-8166-7-4 24410907 PMC3905969

[B48] Xu Y. Chen L. Liu Y. Hao Y. Xu Z. Deng L. (2019). Screening, prenatal diagnosis, and prenatal decision for sex chromosome aneuploidy. Expert Rev. Mol. Diagn. 19, 537–542. 10.1080/14737159.2019.1613154 31081704

[B49] Xu Z. Y. Geng Q. Luo F. W. Xu F. Li P. Xie J. S. (2014b). Multiplex ligationdependent probe amplification and array comparative genomic hybridization analyses for prenatal diagnosis of cytogenomic abnormalities. Mol. Cytogenet. 7, 84. 10.1186/s13039-014-0084-5 25530804 PMC4271441

[B50] Zepeda-Mendoza C. J. Ibn-Salem J. Kammin T. Harris D. J. Rita D. Gripp K. W. (2017). Computational prediction of position effects of apparently balanced human chromosomal rearrangements. Am. J. Hum. Genet. 101, 206–217. 10.1016/j.ajhg.2017.06.011 28735859 PMC5544382

[B51] Zhou Q. H. Wu S. Y. Amato K. Diadamo A. Li P. (2016). Spectrum of cytogenomic abnormalities revealed by array comparative genomic hybridization in products of conception culture failure and normal karyotype samples. J. Genet. Genomics 43, 121–131. 10.1016/j.jgg.2016.02.002 27020032

